# Effect of propranolol on heart rate variability in hyperthyroidism

**DOI:** 10.1186/s13104-018-3224-x

**Published:** 2018-02-22

**Authors:** Aurel T. Tankeu, Marcel Azabji-Kenfack, Chris-Nadège Nganou, Eliane Ngassam, Liliane Kuate-Mfeukeu, Camille Mba, Mesmin Y. Dehayem, Jean-Claude Mbanya, Eugene Sobngwi

**Affiliations:** 1 National Obesity Center, Yaoundé Central Hospital, Yaoundé, Cameroon; 2 Faculty of Medicine and Biomedical Sciences, University of Yaoundé 1, Yaoundé, Cameroon; 3 Cardiology Unit, Yaounde Central Hospital, Yaoundé, Cameroon

**Keywords:** Hyperthyroidism, Heart rate variability, Propranolol, Effect

## Abstract

**Objectives:**

We aimed to determine the effect of propanolol on heart rate variability (HRV) in hyperthyroidism before antithyroid treatment. This was a before and after study, on ten patients presenting overt hyperthyroidism naïve to treatment. In each patient, a resting electrocardiogram was done followed by estimation of cardiac autonomic dysfunction during five maneuvers (Ewing battery tests). Long term HRV measurement was done using 24 h ambulatory electrocardiographic recording. This automatically provided estimation of HRV using SDNN and RMSSD index, LF, HF, and HF/LF ratio. After baseline investigations, 40 mg of propanolol was given twice a day for 3 days and same parameters were measured after 72 h of treatment.

**Results:**

Our patients were aged 40 ± 10 years. Propanolol significantly reduced RR and HR interval (669 ms vs 763 ms and 91 vs 79 bpm; p < 0.01). QT and PR space were significantly extended (360 vs 384 ms and 133 vs 172 ms; p = 0.01). It increases QRS complex and blood pressure response to sustained handgrip but failed to modify previously decreased heart response to deep breathing. HRV parameters such as SDNN, RMSSD, LF, HF and sympathovagal balance estimate by HF/LF ratio remained unchanged. Although a significant reduction in heart excitability, propanolol failed to restore a good sympathovagal balance in hyperthyroidism.

*Trial registration* NCT03393728 “Retrospectively registered”

## Introduction

Typical clinical signs of hyperthyroidism such as increase in heart rate, cardiac output, systolic blood pressure, myocardial contractility, the presence of tremor suggest a hyper adrenergic state and β-adrenergic blocking agents (β-blockers) have been used in this indication for many years as they rapidly improve signs and symptoms observed in patients with thyrotoxicosis [1–4]. Propranolol is widely used in hyperthyroidism to relieve cardiac and peripheral manifestations of thyrotoxicosis and is considered as the drug of choice in the treatment of hyperthyroidism [3, 4]. However, it has been suggested that the beneficial effect of propanolol was not related to a reduction in hormone level suggesting that they must be another mechanism of action [5]. On the other hand, thyrotoxicosis is accompanied by a sympathovagal imbalance characterized by a reduced vagal tone and a subsequent increase in sympathetic activity which may contribute to sinus tachycardia generating some cardiac signs of hyperthyroidism including increase in resting heart rate and arrhythmias [6, 7]. Reduced heart rate variability (HRV), a marker of autonomic dysfunction, predicts mortality after acute myocardial infarction and propanolol has been thought to improve recovery of parasympathetic tone in these patients, thereby reducing risk of sudden death [8]. Evidence indicates that the cardiovascular state due to vagal inhibition in patients suffering from thyroid dysfunctions is poor, an attempt should therefore be made to improve vagal tone especially in hyperthyroid subjects to achieve a stable sympathovagal and cardiovascular homeostasis [9]. Although the peripheral effects of propanolol on the metabolism of thyroid hormones such as reducing peripheral conversion of FT4 to FT3 and antagonizing the β-receptor-mediated effects of catecholamines are well known, many questions on its intra-cardiac effect are still unanswered. Whether β blockers, especially propanolol could influence the improvement HRV in hyperthyroidism is unknown and few data are available on the topic [10]. The purpose of this study was to investigate the effect of propranolol on HRV in patients with hyperthyroidism naive to treatment.

## Main text

### Methods

#### Setting and study population

This was a before and after study, which took place at the National Obesity Center of the Yaoundé Central Hospital. The study population was made up of recently diagnosed patients with hyperthyroidism and naïve to antithyroid treatment. All participants fulfilled diagnosis criteria of thyrotoxicosis. Patients having evidence of cardiopulmonary disease or arrhythmia or presenting a contra-indication to beta blockers were excluded. No other medication was taken during the study period.

#### Procedure and investigations

The study procedure was made of an inclusion visit and three exploratory visits. All patients presenting with signs or symptoms of hyperthyroidism or referred for the management of hyperthyroidism were approached and invited for inclusion.

#### Inclusion visit

The study goal and procedure were explained and eligibility assessed. A cardiovascular exam and a resting electrocardiogram were done to every eligible patient in order to exclude those already presenting with findings which may alter HRV analysis such as arrhythmias, bundle branch blocks and myocardial infarction and overt cardiovascular abnormalities.

#### Exploratory visits

At the first exploration visit, a resting electrocardiogram was done to every patient. This was followed by the measure of HRV using a continuous electrocardiogram device (heart rate meter Polar RS 800). Electrodes were moisten and placed at the level of the 5th rib on the chest wall in order to pick up waves coming from heart beats and the corresponding watch used to record data attached at the left wrist. This device helped in the evaluation of HRV during a battery of tests and on the following 24 h.

In order to evaluate cardiac autonomic function in our population study, every patient had to performed a battery of five tests according to Ewing battery tests used to assess cardiac autonomic dysfunction [11]. Five minutes after the end of the last maneuver, the patient returned home with the device functioning for the following 24 h. An appointment was given 24 h later and patient went back with the device recording heart rate all day long.

The following day, the device was stopped and each patient received 80 mg of propranolol per day for 03 days administered as 40 mg every 12 h. The next appointment was given 03 days later. The drug was stopped and another resting electrocardiogram was done considering the same measures. HRV was recorded for the second time during the Ewing battery tests and for 24 h using the same procedure.

#### Statistical analysis

Data acquisition and analysis were performed using SPSS version 13.0. Continuous variables are expressed as means with standard deviation and categorical variables as count. The Wilcoxon test was used to test differences between before and after treatment measurements. A p < 0.05 was considered as statistically significant.

### Results

#### General characteristics

We enrolled 10 patients presenting overt hyperthyroidism (9 females) with an average age of 40 ± 10 years with extremes ranging from 27 to 60 years. Mean blood pressure of the study population was 115/62 mmHg for a mean heart rate of 98 ± 20 beats/min. The analysis of ECG showed that all ECG parameters were improve after propanolol administration (Table 1).

**Table 1:** Comparison of ECG parameters before and after propanolol

Variables	Before	After	p (Wilcoxon)
HR	91 ± 18	79 ± 9	0.02
R–R	668.8 ± 126.8	762.4 ± 77.3	0.01
QT	360.9 ± 29.2	384.3 ± 15.9	0.01
Corrected QT	447 ± 10.9	440.8 ± 21.5	0.86
P–R	133.9 ± 54.7	172.7 ± 43.63	0.01
QRS	93 ± 6.1	97 ± 10.2	0.01
Mean RR (s)	0.71 ± 0.08	0.79 ± 0.06	0.02
Mean HR (min^−1^)	85.5 ± 9.3	76.1 ± 5.1	0.02

HRV evaluate by the Ewing battery tests showed abnormal results for the second and the fifth tests at baseline before propanolol administration. Propanolol administration failed to modify heart response to deep breathing but significantly increased blood pressure response to sustained handgrip (− 6.8 vs 1.2; p = 0.04). However, that increase, although significant, remained under normal values set to ≥ 16 (Table 2).

**Table 2:** Comparison of Ewing tests results before and after propanolol

Test	Before	After	p
Valsalva maneuver	1.27 ± 0.14	1.20 ± 0.10	0.09
HR to standing	1.18 ± 0.1	1.26 ± 0.24	0.39
HR response to deep breathing	10.6 ± 4.5	11.17 ± 2.80	0.61
BP response to standing	2 ± 4.7	1.20 ± 3	0.23
BP response to sustained handgrip	− 6.8 ± 6.3	1.20 ± 3	0.04

The average value of the R-R interval was significantly increased by propranolol (0.71 vs 0.79; p = 0.028). In contrast, SDNN index, representing the overall HRV but mostly the sympathetic tone remained unchanged after propanolol administration as well as RMSSD, a marker of parasympathetic activity. Otherwise the LF (n.u.), HF (n.u.) and LF/HF ratio were not significantly changed after the administration of propranolol in our population study (Table 3).

**Table 3:** Comparison of HRV before and after propanolol

Variable	Before	After	p
RMSSD (ms)	22.3 ± 13.4	25.2 ± 12.1	0.17
SDNN index	34.1 ± 14.6	35.8 ± 11.7	0.75
LF (n.u.)	51.2 ± 24.5	50.8 ± 22	0.91
HF (n.u.)	28.6 ± 19.5	31.9 ± 18.6	0.46
LF/HF	4.3 ± 5.6	3.1 ± 3.2	0.24

*RR* R to R interval, *HR* heart rate, *RMSSD* root-mean-square of successive differences, *SDNN* standard deviation of all normal R to R interval, *LF* low frequency, *HF* high frequency

#### Adverse events

None.

### Discussion

This study was carried out in order to determine the effect of propanolol administration on HRV in patients with hyperthyroidism naïve to antithyroid treatment. Our results indicate that 80 mg of propanolol taken for 03 days can significantly slow heart rate and modify parameters of resting ECG. Despite this reduction in cardiac excitability and nerve impulse conduction, it failed to increase parasympathetic tone and overall HRV. It can slightly increase blood pressure response to sustained handgrip though this increase remains under normal set values. A previous study showed that propanolol is effective in the recovery of HRV in patients after 6 weeks of treatment following an acute myocardial infarction [8]. The absence of effect in our study could either be attributed to the short duration of treatment or to an absence of modification specific to HRV in hyperthyroidism. The same beneficial effect of beta-blockers was found in rats, healthy volunteers and patients with ESKD using either lipophilic or hydrophilic beta-blockers which appeared to be effective in increasing the vagal modulation of heart rate [12–15]. Another reason that could explain the absence of effect in our study is the lipophilicity of propanolol. A comparative evaluation of hydrophilic and lipophilic effects of beta-blockers on HRV suggested that hydrophilic drug achieving peripheral blockade of beta-adrenergic receptors provided greater improvement in HRV than lipophilic drugs and this was associated with a higher HF power and a lower LF/HF ratio during day time activity and stress [16].

To summarize, the reduction of sinus tachycardia obtained by short term treatment with propanolol in hyperthyroidism may be independent of modification of HRV. This suggests that the reduction of HRV found in hyperthyroidism by many studies is not of great participation in heart rate modification and cardiac signs observed in this condition since propanolol which is one of the most effective drug used in this indication did not significantly modify these parameters despite the clinical effect.

### Limitations

Our study was not a double-blind, placebo-controlled randomized trial what would best evaluate an existing effect.

## Abbreviations

BP: blood pressure
HR: heart rate
HRV: heart rate variability
HF: high frequency
LF: low frequency
ms: milliseconds
RMSSD: root-mean-square of successive differences
SDNN: standard deviation of all normal R–R intervals
TSH: thyroid stimulating hormone
